# Pgp3 seroprevalence and associations with active trachoma and ocular *Chlamydia trachomatis* infection in Malawi: cross-sectional surveys in six evaluation units

**DOI:** 10.1371/journal.pntd.0007749

**Published:** 2019-10-28

**Authors:** Sarah E. Burr, John Hart, Lyson Samikwa, David Chaima, Gretchen Cooley, Diana Martin, Michael Masika, Anthony W. Solomon, Robin L. Bailey, Khumbo Kalua

**Affiliations:** 1 Department of Clinical Research, London School of Hygiene & Tropical Medicine, London, United Kingdom; 2 College of Medicine, University of Malawi, Blantyre, Malawi; 3 Division of Parasitic Diseases and Malaria, Centers for Disease Control and Prevention, Atlanta, Georgia, United States of America; 4 Ministry of Health, Lilongwe, Malawi; 5 Blantyre Institute for Community Outreach, Blantyre, Malawi; RTI International, UNITED REPUBLIC OF TANZANIA

## Abstract

**Background:**

Following one to five years of antibiotic mass drug administration (MDA) for the elimination of trachoma as a public health problem, programmes must conduct impact surveys to inform decisions on whether MDA is still needed. These decisions are currently based on the prevalence of trachomatous inflammation—follicular (TF), which, after MDA, correlates poorly with prevalence of ocular *Chlamydia trachomatis* infection.

**Methodology/Principal findings:**

Impact surveys in six evaluation units (EUs) of Malawi were used as a platform to explore associations between the prevalence of TF, ocular *C*. *trachomatis* infection and anti-Pgp3 antibodies one year after the third annual round of MDA. Participants were examined for trachoma using the World Health Organization simplified grading system. Ocular swabs and dried blood spots (DBS) were collected from children aged 1–9 years. Swabs were tested for *C*. *trachomatis* DNA using GeneXpert. DBS were assayed for anti-Pgp3 antibodies using ELISA. EU-level prevalence of TF in children aged 1–9 years ranged from 4.7% (95% CI 3.4–6.3) to 7.2% (95% CI 5.8–8.9). Prevalence of *C*. *trachomatis* infection in children ranged from 0.1% (95% CI 0.0–0.6) to 0.7% (95% CI 0.3–1.3) while Pgp3 seroprevalence ranged from 6.9% (95% CI 5.4–8.6) to 12.0% (95% CI 10.1–14.0) and increased with age.

**Conclusions/Significance:**

Based on current global policy, the prevalence of TF indicates that a further year of antibiotic MDA is warranted in four of six EUs yet the very low levels of infection cast doubt on the universal applicability of TF-based cut-offs for antibiotic MDA. Pgp3 seroprevalence was similar to that reported following MDA in other settings that have reached the elimination target however the predictive value of any particular level of seropositivity with respect to risk of subsequent infection recrudescence is, as yet, unknown.

## Author summary

Trachoma, caused by ocular infection with the bacterium *Chlamydia trachomatis*, is treated at the population level with mass drug administration (MDA) of azithromycin, a broad-spectrum antibiotic. Decisions to stop MDA are subsequently made following impact surveys designed to determine the prevalence of trachoma in children aged 1–9 years. However, clinical signs of trachoma can persist in treated communities even in the absence of active *C*. *trachomatis* infection, potentially leading to continued MDA that may be unnecessary. In this study, we use the platform provided by six impact surveys to compare prevalence of clinical signs of trachoma in children with prevalence of *C*. *trachomatis* infection and anti-*C*. *trachomatis* antibodies. Based on current policy, prevalence of clinical signs of trachoma in four of six evaluation units surveyed indicated further MDA is warranted. However, tests for *C*. *trachomatis* DNA indicate extremely low levels of infection making the need for further MDA unclear. Prevalence of anti-*C*. *trachomatis* antibodies were similar to that found in other low prevalence settings, however the significance of such levels, and of levels of current *C*. *trachomatis* infection, with regard to the risk of reinfection are, as yet, unknown.

## Introduction

Trachoma, caused by repeated infection with the obligate intracellular bacterium *Chlamydia trachomatis*, is a leading cause of preventable blindness. Elimination of trachoma as a public health problem can be achieved through implementation of the multi-faceted SAFE strategy: Surgery to correct trichiasis, Antibiotics to treat ocular *C*. *trachomatis* infection, and Facial cleanliness and Environmental improvement to reduce transmission [1–2].

The A, or antibiotic, component of SAFE is carried out at the district level through mass drug administration (MDA) of the macrolide antibiotic azithromycin. Prevalence of trachomatous inflammation—follicular (TF) in children aged 1–9 years currently guides MDA decision-making. One to five years of district-level MDA is undertaken wherever baseline prevalence of TF in children aged 1–9 years is ≥5%. The decision to stop MDA is again based on the prevalence of TF, determined through adequately powered impact surveys [3]. If the TF prevalence in children aged 1–9 years remains ≥5%, district-level MDA may continue.

The use of TF as the sole indicator to guide decisions on whether to continue or cease MDA following impact surveys may be problematic because low levels of TF can persist in the absence of ocular *C*. *trachomatis* infection [4]. Follicular conjunctivitis can be associated with non-chlamydial bacterial infection [5–6] or be of uncertain aetiology, even in apparently trachoma-endemic populations [7]. Furthermore, as active trachoma prevalence declines, it becomes increasingly difficult to train graders for surveys and to prove that graders have been trained well [8]. These issues have led to suggestions that alternate indicators for use in MDA decision-making and post-MDA surveillance be explored [9–10]. Possible alternatives include nucleic acid amplification-based tests for ocular *C*. *trachomatis* infection and assays for the presence of anti-*C*. *trachomatis* antibodies [11–12].

Malawi represents an ideal setting in which to assess the utility of alternate indicators for trachoma elimination programmes. Health officials have known Malawi to be endemic for trachoma since the 1980s [13] although an active control programme was not put in place until a decade ago. The Trachoma Action Plan was developed and launched by the Ministry of Health in 2011. The Global Trachoma Mapping Project completed mapping trachoma in Malawi in 2015 and showed, in combination with data collected previously, that 17 of 28 districts had TF prevalence between 10 and 25% [14–16]. Funding to implement the SAFE strategy in these 17 districts was made possible through the Queen Elizabeth Diamond Jubilee Trust in 2014, with the goal of eliminating trachoma by 2019. Several districts have since completed three rounds of MDA with high treatment coverage [17] and have proceeded to impact surveys, following WHO guidelines. To date, approximately half the population has received MDA and six thousand individuals have received surgery for trichiasis. The aim of the present study was to generate, in a programmatic setting, additional data on *C*. *trachomatis* infection and anti-Pgp3 antibodies as possible alternative indicators for making decisions as to whether or not to stop MDA.

## Methods

### Study site

The study was conducted in two districts: Chikwawa in southern Malawi (estimated population 565,000) [18] and Mchinji (est. pop. 600,000) [18] in central Malawi. Trachoma prevalence was previously assessed in both districts through surveys conducted in 2008 [14]; the prevalence of TF in children aged 1–9 years was 13.6% in Chikwawa and 21.7% in Mchinji. Prevalence of trichiasis in adults aged ≥15 years was 0.6% (Chikwawa) and 0.3% (Mchinji) [14]. Following the 2008 surveys, a trachoma elimination programme was devised and, as a part of that programme, both districts received azithromycin MDA at yearly intervals for three years (2011–2013).

### Sampling strategy

For the purposes of the impact surveys, following WHO guidance, each district (Chikwawa and Mchinji) was divided into three evaluation units (EUs) [3]. To estimate a prevalence of TF, in children aged 1–9 years, of 10% in each EU, given a desired precision of ±3%, a 95% confidence limit and a design effect of 2.65, 1,019 children should be examined [8]. The average Malawian household was estimated to contain 4 to 5 people, of who 1–2 are in the 1–9 years age range. Therefore, to achieve the required sample size, taking into account non-response, 24 clusters (villages) were randomly selected in each EU and 30 households were randomly selected, from a list of households, in each cluster. Every consenting member of each selected household aged ≥1 year was examined.

### Data collection

Data collection took place in June and July 2014. Prior to the onset of fieldwork, a workshop was held for trachoma graders (ophthalmic clinical officers) and enumerators. Training included quality control exercises of trachoma grading from slides in a classroom setting followed by practical tests in the field [19].

Questionnaires were used to capture demographic information and data on household-level access to water and sanitation. For the latter, questions were asked of the head of each selected household, and (where relevant) sanitation facilities observed. All household members, defined as those who slept in the household the night before, were eligible for trachoma screening. Consenting individuals were examined for clinical signs of trachoma using a 2.5× magnifying loupe and adequate sun- or torchlight. Trachoma grading was carried out according to the WHO simplified grading system [20].

Ocular swabs and dried blood spots (DBS) were collected from children aged 1–9 years. Ocular samples were collected using Dacron swabs (Puritan, Guilford, USA) passed across the upper left tarsal conjunctiva, after everting the eyelid. Field control swabs were also taken, once for every 50 swabs collected (with timing determined at random), by passing a clean swab in the air within two inches of a child’s eyes. All swabs were placed in sterile tubes and kept on cool packs in the field and frozen at -20°C within 10 hours of collection. Blood samples were collected by finger prick with a new, sterile lancet, and absorbed onto filter papers calibrated to absorb 10 μL of blood (TropBio, Townsville, Australia). These were then air-dried, sealed in individual plastic bags and placed in larger bags with desiccant before being stored at -20°C.

### Laboratory methods

Swabs were tested for *C*. *trachomatis* DNA using the GeneXpert CT/NG assay (Cepheid Inc., Sunnyvale, CA, USA). Swabs were rehydrated in 800 μL nuclease-free water and vortexed vigorously. Two hundred μL of suspension from each of five swabs was then combined to generate a pool [21]. Three hundred μL of the pool was added to 900 μL of CT/NG transport media (Cepheid Inc.) and this total volume added to a GeneXpert CT/NG cartridge (Cepheid Inc.) and tested according to the manufacturer’s instructions. In the case of a positive or invalid result, all five samples making up the pool were then tested individually. Known positive and negative controls, supplied by the London School of Hygiene & Tropical Medicine (London, UK), were run every week during testing as a quality control measure.

Laboratory personnel were blind to the clinical status of subjects from whom samples were taken and were unable to distinguish between ocular swabs and swabs included as field controls.

DBS were tested, in duplicate, for antibodies against Pgp3 [22] according to the method previously described [11, 22]. Serum was eluted overnight then applied to Immulon 2HB microtiter plates (Southern Biotech, Birmingham, AL, USA) pre-sensitized with Pgp3 protein. Bound antibody was detected with HRP-labelled mouse anti-human IgG (Fc)-HRP (Southern Biotech). Plates were incubated, washed and 3,3’,5,5’-tetramethylbenzidine (KPL, Gaithersburg, MD, USA) was added before the reaction was stopped with 1N H_2_SO_4_. Absorbance was read at 450 nm (A_450_) using a HaloLED 96 Microplate Reader (Dynamica, London, UK). Readings were corrected for background by subtracting the average A_450_ of two blank wells containing no serum. Values were normalised by dividing the mean of values for the two wells against the mean for the two 200 U controls included on each plate [23].

### Data entry and statistical analysis

All field data were collected using an Android smartphone app and uploaded to a password-protected cloud-based server. Laboratory data were captured electronically on the GeneXpert and HALO plate reader devices.

Data were analyzed using Stata version 14.2 (StataCorp LP, College Station, TX, USA). Prevalence of TF, *C*. *trachomatis* infection and antibodies to Pgp3 were calculated and displayed with exact binomial confidence intervals. Correlations between EU-summarised prevalence of TF, *C*. *trachomatis* infection and antibodies to Pgp3 were plotted with lines of best-fit and tested using Spearman’s rank correlation. Cluster-summarized prevalence of the three indicators was mapped using ArcMap v10.3 (Environmental Systems Research Institute, Inc. Redlands, CA, USA).

A finite mixture model was used to classify the samples as seropositive or seronegative based on normalized A_450_ values [22]. The cutoff for seropositivity was defined as the mean of the Gaussian distribution of the seronegative population plus three standard deviations of the seronegative population [22].

Seroconversion rates (SCR) were calculated by fitting a simple reversible catalytic model to the measured seroprevalence, stratified into yearly age-groups, using maximum likelihood methods, assuming no seroreversion [24].

### Case management

All individuals with TF were provided with two tubes of 1% tetracycline ointment and instructions for twice-daily use for six weeks. Cases of trichiasis were referred for surgery.

### Ethics statement

The study adhered to the tenets of the Declaration of Helsinki and was approved by Malawi’s Ministry of Health National Health Sciences Ethics and Research Committee. Written, informed consent was obtained from the parent or guardian of all children. In the case of adults whose eyes were examined but from whom no samples were taken, verbal consent was obtained and documented electronically. CDC staff did not have contact with study participants and were determined to be non-engaged in the study.

## Results

Overall, 13,556 individuals, representing 94.3% of the censused population, participated in the study. Of these, 6,314 (43.9%) were children aged 1–9 years. Demographic characteristics of individuals and households are given in Table 1.

**Table 1:** Characteristics of study participants.

Characteristic	No. (%)
**Total censused population**	14,379
Gender	
Male	6,097 (42.4)
Female	8,282 (57.6)
Age (years)	
1–9	6,314 (43.9)
10–14	1,452 (10.1)
≥15	6,613 (46.0)
**Total population examined**	
Yes	13,556 (94.3)
No–absent	771 (5.4)
No–refused	48 (0.3)
No–other	4 (0.0)
**Household characteristics**	
Main source of drinking water	
Piped water into dwelling	62 (0.4)
Piped water into yard	165 (1.2)
Public tap	1,400 (9.7)
Borehole	9,920 (69.0)
Protected dug well	552 (3.8)
Unprotected dug well	1,698 (11.8)
Unprotected spring	175 (1.2)
Surface water	382 (2.7)
Other	25 (0.2)
Habitual time to fetch water	
Water source in yard	420 (2.9)
Less than 30 minutes	10,081 (70.1)
30 minutes to 1 hour	3,232 (22.5)
More than 1 hour	646 (4.5)
Access to latrine	
Shared or public latrine	3,513 (24.4)
Private latrine	9,686 (67.4)
No structure, outside house	106 (0.7)
No structure, in bush	1,066 (7.4)
Other	8 (0.1)
Type of latrine (observed)	
Flush/pour	86 (0.5)
Ventilated, improved pit latrine	33 (0.2)
Pit latrine	13,221 (91.9)
No facilities, bush or field	1,036 (7.2)
Other	23 (0.2)

The majority of households reported accessing their drinking water at boreholes (69%) and 70% of households were reportedly within 30 minutes of their water source (Table 1). Access to sanitation was good, with over 90% of households reporting access to either shared or private latrines (Table 1).

District-level prevalence of trichiasis in participants 15 years of age and older was 0.43% in Chikwawa (13/2,999) and 0.12% (4/3,251) in Mchinji. There was no difference in the prevalence of TT amongst female (11/4,179; 0.26%) and male (6/2,071; 0.29%) participants.

Prevalence of TF amongst children aged 1–9 years ranged from 4.7% (95% CI 3.4–6.3) in the EU of Kasisi/DHO to 7.2% (95% CI 5.8–8.9) in the EU of Makanda Gumba (Table 2). Overall TF prevalence in children aged 1–9 years was 5.1% (95% CI 4.3–6.0) in Chikwawa district and 6.4% (95% CI 5.6–7.3) in Mchinji (Table 2).

**Table 2:** Prevalence of trachomatous inflammation—follicular (TF), *C*. *trachomatis* infection and anti-Pgp3 antibodies in participants aged 1–9 years.

	TF				Ocular Ct infection				Anti-Pgp3 antibodies			
	N	n	%	95% CI^1^	N	n	%	95% CI^1^	N	N	%	95% CI^1^
**Overall**	6,075	354	5.8	5.3–6.4	5,866	18	0.3	0.2–0.5	5,917	570	9.6	8.9–10.4
**EU**												
Chapananga^2^	937	46	4.9	3.6–6.5	924	2	0.2	0.0–0.8	916	104	11.4	9.4–13.6
Ngabu Ngokwe^2^	914	52	5.7	4.3–7.4	881	1	0.1	0.0–0.6	897	88	9.8	7.9–11.9
Kasisi/DHO^2^	869	41	4.7	3.4–6.3	863	2	0.2	0.0–0.8	830	67	8.1	6.3–10.1
Makanda Gumba^3^	1,120	81	7.2	5.8–8.9	1,085	7	0.7	0.3–1.3	1,117	134	12.0	10.1–14.0
Luzi Kochilira^3^	1,183	77	6.5	5.2–8.1	1,119	3	0.3	0.1–0.8	1,139	107	9.4	7.8–11.2
DHO Nkwazi^3^	1,052	57	5.4	4.1–7.0	994	3	0.3	0.1–0.9	1,018	70	6.9	5.4–8.6
**District**												
Chikwawa	2,720	139	5.1	4.3–6.0	2,668	5	0.2	0.1–0.4	2,643	259	9.8	8.7–11.0
Mchinji	3,355	215	6.4	5.6–7.3	3,198	13	0.4	0.2–0.7	3,274	311	9.5	8.5–10.6
**Gender**												
Female	3,151	192	6.1	5.3–7.0	3,033	12	0.4	0.2–0.7	3,076	293	9.5	8.5–10.6
Male	2,924	162	5.5	4.7–6.4	2,833	6	0.2	0.1–0.5	2,841	277	9.8	8.7–10.9
**Age (years)**												
1	650	37	5.7	4.0–7.8	626	2	0.3	0.0–1.1	637	24	3.8	2.4–5.6
2	910	85	9.3	7.5–11.4	852	2	0.2	0.0–0.8	882	39	4.4	3.2–6.0
3	800	55	6.9	5.2–8.9	765	2	0.3	0.0–0.9	780	56	7.2	5.5–9.2
4	738	53	7.2	5.4–9.3	710	1	0.1	0.0–0.8	716	48	6.7	5.0–8.8
5	724	39	5.4	3.9–7.3	703	2	0.3	0.0–1.0	708	64	9.0	7.0–11.4
6	603	43	7.1	5.2–9.5	589	2	0.3	0.0–1.2	591	73	12.4	9.8–15.3
7	596	20	3.4	2.0–5.1	585	4	0.7	0.2–1.7	581	76	13.1	10.4–16.1
8	453	13	2.9	1.5–4.9	442	2	0.5	0.1–1.6	436	73	16.7	13.4–20.6
9	601	9	1.5	0.7–2.8	594	1	0.2	0.0–0.9	586	117	20.0	16.8–23.4

^1^Exact binomial confidence interval

^2^EU in Chikwawa

^3^EU in Mchinji

Ocular swabs were collected from 6,075 children. Of these, 185 ocular swabs gave invalid results, indicating they did not contain detectable amounts of human DNA. Swabs from 24 children were missing. A positive or negative PCR result was obtained for the remaining 5,866 swabs (96.6% of the total). The prevalence of *C*. *trachomatis* infection was low, ranging from 0.1% (95% CI 0.0–0.6) in the EU of Ngabu Ngokwe to 0.7% in Makanda Gumba (95% CI 0.3–1.3) (Table 2). District-level prevalence of infection was 0.2% (95% CI 0.1–0.4) in Chikwawa and 0.4% (95% CI 0.2–0.7) in Mchinji (Table 2). Of the 264 negative field controls tested, none gave a positive result for *C*. *trachomatis* DNA.

Pgp3 ELISA results were obtained for 97.4% of recruited children (5,917/6,075) aged 1–9 years. The mean absorbance value was 0.3581 (standard deviation 0.2765). Seroprevalence was lowest in DHO Nkwazi [6.9% (95% CI 5.4–8.6)] and highest in Makanda Gumba [12.0% (95% CI 10.1–14.0)] and increased with age. The lowest seroprevalence levels were found in children aged 1 year [3.8%, (95% CI 2.4–5.6)] while the highest levels were in children aged 9 years [20.0%, (95% CI 16.8–23.4)] (Table 2). Seroprevalence was similar in both districts; 9.8% (95% CI 8.7–11.0) in Chikwawa and 9.5% (95% CI 8.5–10.6) in Mchinji (Table 2). The seroconversion rate (SCR) was 0.0283 (95% CI: 0.0275–0.029) per year for Chikwawa and 0.021 (95% CI: 0.020–0.021) for Mchinji.

**Fig 1:**
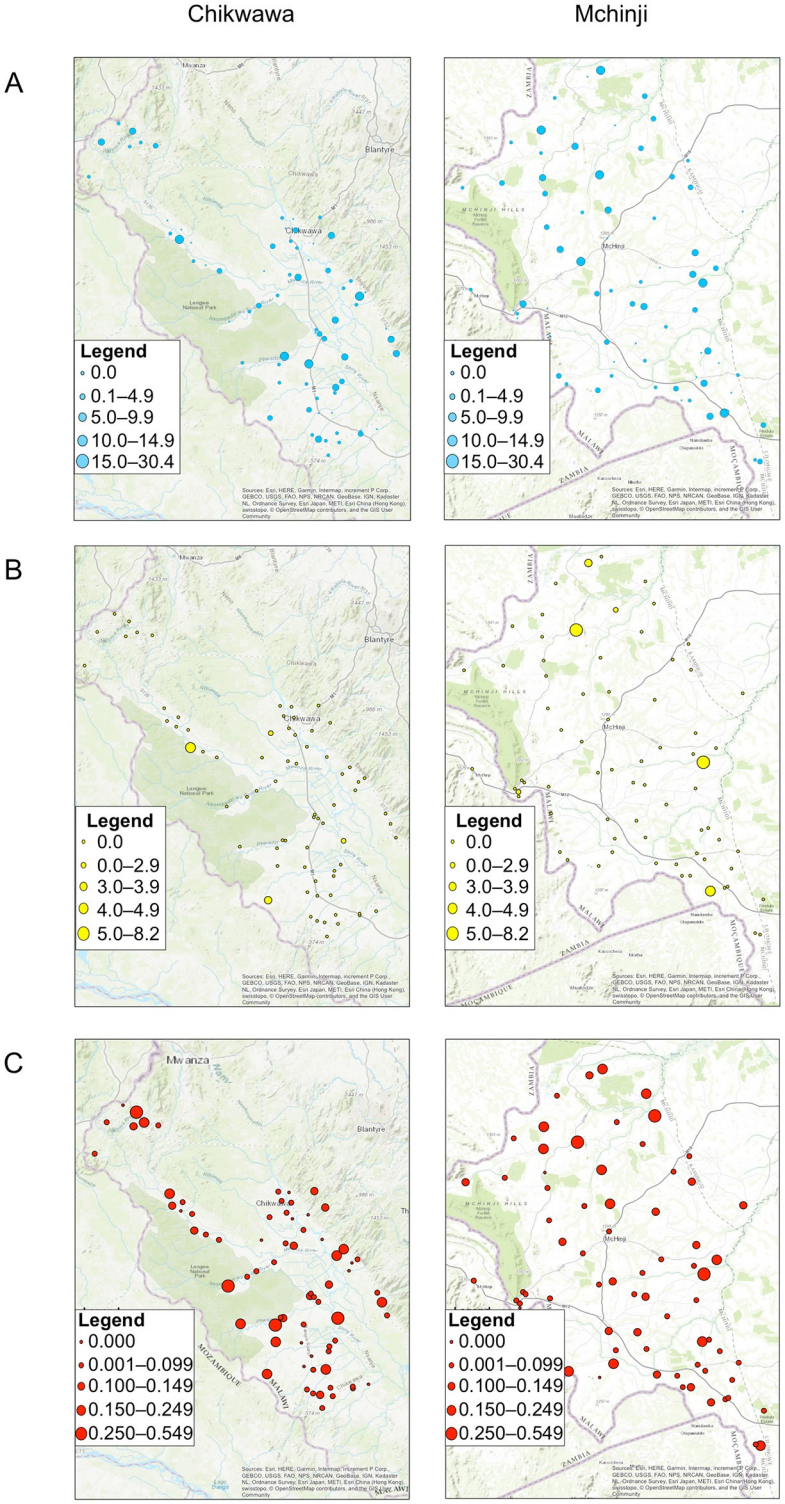
Mapping of indicators of trachoma in Chikwawa and Mchinji. Cluster level prevalence of (A) TF, (B) *C*. *trachomatis* infection and (C) anti-Pgp3 antibodies.

Geographical distributions of TF, *C*. *trachomatis* infection and prevalence of anti-Pgp3 antibodies in children aged 1–9 years are shown in Fig 1. The highest prevalence of all three indicators were found in the EU of Makanda Gumba (Table 2).

**Fig 2:**
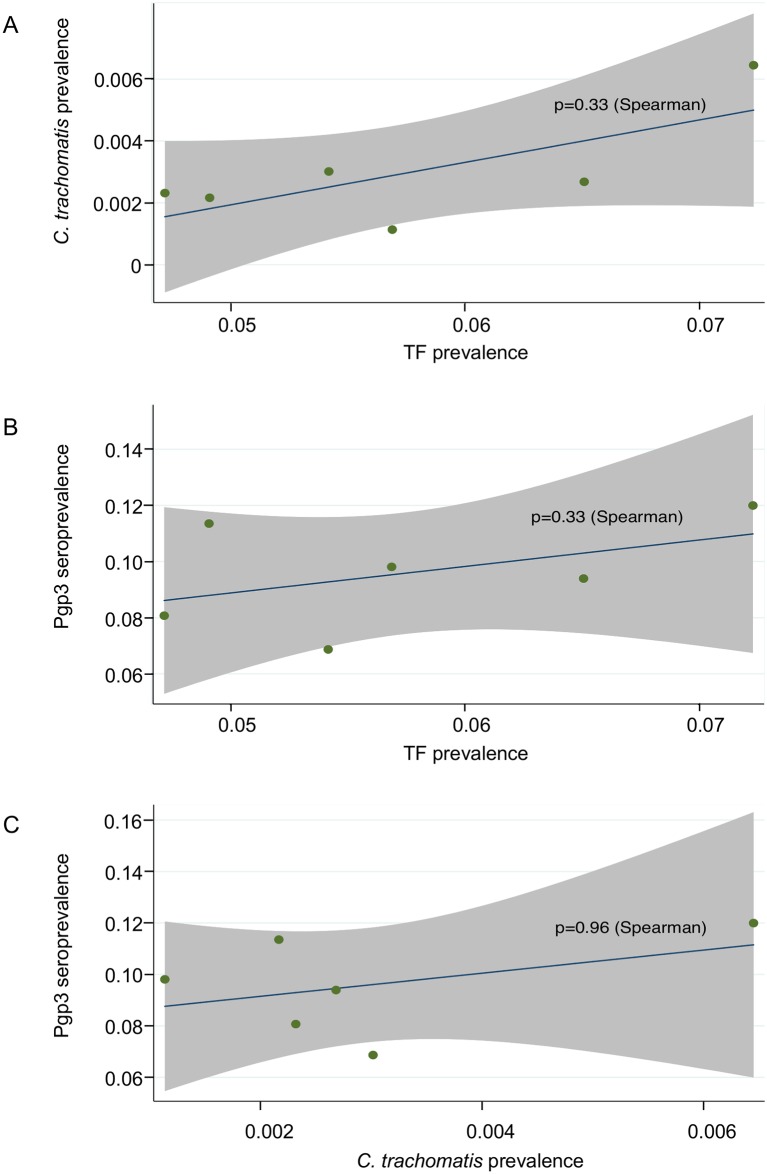
Correlations between EU-summarised prevalence of TF, *C*. *trachomatis* infection and anti-Pgp3 antibodies. (A) Correlation of EU-summarised prevalence of TF and *C*. *trachomatis* infection. (B) Correlation of EU-summarised prevalence of TF and anti-Pgp3 antibodies. (C) Correlation of EU-summarised prevalence of *C*. *trachomatis* infection and anti-Pgp3 antibodies. EUs are represented by green dots. Lines of best fit are shown in blue. Shaded areas denote 95% confidence intervals.

No association was seen between any of EU-summarised prevalence of TF and *C*. *trachomatis* infection (Fig 2A), EU-summarised prevalence of TF and anti-Pgp3 antibodies and EU-summarised prevalence of *C*. *trachomatis* infection and anti-Pgp3 antibodies (Fig 2C).

## Discussion

Our data show that prevalence of TF one year following the completion of three annual rounds of MDA was low in all six EUs surveyed. Two EUs have reached the elimination TF prevalence threshold of less than 5% in children aged 1–9 years [25] while the remaining four EUs are nearing this target. The prevalence of ocular *C*. *trachomatis* infection in the same children was 0.3%, considerably lower than that of active disease. The persistence of low-level rates of TF in communities where ocular infection is rare has previously been clearly documented in trachoma-endemic populations approaching elimination [4] and has been responsible, in part, for prompting the discussion of whether TF is the most appropriate marker on which to base decisions to stop MDA.

According to current guidelines, antibiotic MDA should be stopped only when impact surveys in EUs (population 100,000–250,000 people) indicate the prevalence of TF in children aged 1–9 years is below 5% [25]. The present results therefore indicate that another year of antibiotic MDA is warranted in four of the six EUs before re-survey. Yet, when viewed alongside PCR data that indicate a virtual absence of *C*. *trachomatis* infection [prevalence 0.3% (95% CI 0.2–0.5)], doubt is cast on the need for further antibiotic use. There is, however, no current recommendation for programmatic application of data on infection and PCR testing remains expensive. Serology, which can be conducted at much lower cost and multiplexed for integrated surveillance of multiple infectious diseases [10], may prove a viable alternate indicator for stopping MDA and undertaking post-MDA surveillance.

Overall seroprevalence in the current study (9.6%) was somewhat higher than prevalence of TF (5.8%), which is consistent with the notion that anti-Pgp3 antibody responses are markers of cumulative exposure rather than of persistent infection [26–27]. Furthermore, there was an increase in seropositivity with age seen in the current study, which also reflects cumulative exposure.

The cut-off for determining seropositivity with the ELISA assay used in this study was calculated using the finite mixture model, following the approach recommended by Migchelsen *et al*. [22]. This approach may over-estimate prevalence relative to other methods, such as the receiver operating characteristic (ROC) curves [22–23] used elsewhere [28–30]. Comparison to other studies is further complicated by the varying means of detecting Pgp3 antibodies. Many studies have used bead-based immunoassays [12, 26–27, 30–31], which are more expensive and technically challenging than the ELISA-based method employed here. However, a direct comparison of the two assays (EISA versus bead-based immunoassay) in a pre-validation survey in Ghana showed similar population-level prevalence [5.5% (95% CI: 4.8–6.3) for ELISA; 4.3% (95% CI: 3.7–4.9) for bead-based assay] [32] with calculated SCRs being virtually identical [0.013 (95% CI: 0.011–0.016) for ELISA; 0.012 (95% CI: 0.009–0.016) for bead-based assay). While further direct comparisons of platforms in large-scale surveys are needed, initial studies suggest good agreement of data from different assay platforms, especially if the SCR is the output of greatest interest.

Before serology could be adopted as an alternative or adjunct to TF prevalence for stopping MDA and post-MDA decision-making, seropositivity thresholds would need to be established. One approach might be to focus on the youngest children–those born during MDA or after its cessation and presumably very infrequently exposed to ocular *C*. *trachomatis* infection. Because the current survey was conducted in the year following completion of the third round of MDA and because children under 1 year of age, who might have had transplacentally-transferred maternal antibodies, were not eligible for inclusion, we do not have data from children born after the cessation of MDA. The youngest children in this study (aged 1–3 years) were born during the three-year MDA campaign and had similar seroprevalence to 1–3-year-olds in elimination settings; in post-validation settings, 1–3-year-olds are reported to have a prevalence of anti-Pgp3 antibodies of <6%, as measured primarily by bead-based assays [12, 32–34].

A second approach would be to look at overall seroprevalence in 1–9-year-olds. While the prevalence of anti-Pgp3 antibodies here was much lower than that in populations that have high transmission rates [27–28], the antibody prevalence was higher than in communities where longitudinal follow-up and tests for infection have documented sustained elimination of ocular *C*. *trachomatis* infection [12, 29–30]. For example, a study conducted 10 years after cessation of antibiotic MDA in Rombo district of Tanzania found just 3.5% (95% CI: 1.4–7.1) of 1–9-year-olds to have anti-Pgp3 antibodies [12], less than the 9.6% seropositivity (95% CI: 8.9–10.4) seen in the children in the current study. In The Gambia, 4.2% (95% CI: 2.9–5.9) of children aged 1–9 years were positive for Pgp3 antibodies 5 years after MDA [29], while in Nepal 3.4% (95% CI: 1.4–6.9) of children aged 9 years were seropositive 4 years following cessation of MDA [30].

A third approach would be to look at trends in antibody responses with age. The SCR estimated for the two districts here [0.0283 (95% CI: 0.0275–0.029) per year for Chikwawa and 0.021 (95% CI: 0.020–0.021) for Mchinji] were higher than that predicted to be associated with <5% TF [SCR of 0.015 (95% CI: 0.0–049)] [35], but the confidence intervals were overlapping. An algorithm combining these two metrics–seroprevalence and SCR–may have utility as well, particularly in settings like the Malawi districts studied here, where SCR estimates are close to but not below predictions for TF<5%. While it is unclear that this level of seropositivity is definitively associated with local elimination, the current data add to the evidence base that will be needed to determine appropriate thresholds for potential use of serology for surveillance.

## Supporting information

S1 Data Data set. (CSV)

S2 Data Data dictionary. (CSV)

S1 Checklist STROBE checklist. (DOC)
